# Ion diffusion may introduce spurious current sources in current-source density (CSD) analysis

**DOI:** 10.1152/jn.00976.2016

**Published:** 2017-03-15

**Authors:** Geir Halnes, Tuomo Mäki-Marttunen, Klas H. Pettersen, Ole A. Andreassen, Gaute T. Einevoll

**Affiliations:** ^1^Faculty for Science and Technology, Norwegian University of Life Sciences, Ås, Norway;; ^2^NORMENT, KG Jebsen Centre for Psychosis Research, Institute of Clinical Medicine, University of Oslo, Oslo, Norway;; ^3^Letten Centre and GliaLab, Institute of Basic Medical Sciences, University of Oslo, Oslo, Norway; and; ^4^Department of Physics, University of Oslo, Oslo, Norway

**Keywords:** current source density, electrodiffusion, extracellular potential, ion diffusion

## Abstract

Current-source density (CSD) analysis is a well-established method for analyzing recorded local field potentials (LFPs), that is, the low-frequency part of extracellular potentials. Standard CSD theory is based on the assumption that all extracellular currents are purely ohmic, and thus neglects the possible impact from ionic diffusion on recorded potentials. However, it has previously been shown that in physiological conditions with large ion-concentration gradients, diffusive currents can evoke slow shifts in extracellular potentials. Using computer simulations, we here show that diffusion-evoked potential shifts can introduce errors in standard CSD analysis, and can lead to prediction of spurious current sources. Further, we here show that the diffusion-evoked prediction errors can be removed by using an improved CSD estimator which accounts for concentration-dependent effects.

**NEW & NOTEWORTHY** Standard CSD analysis does not account for ionic diffusion. Using biophysically realistic computer simulations, we show that unaccounted-for diffusive currents can lead to the prediction of spurious current sources. This finding may be of strong interest for in vivo electrophysiologists doing extracellular recordings in general, and CSD analysis in particular.

## Abstract (precis)

Standard CSD analysis does not account for ionic diffusion. Using biophysically realistic computer simulations, we show that unaccounted-for diffusive currents can lead to the prediction of spurious current sources. This finding may be of strong interest for in vivo electrophysiologists doing extracellular recordings in general, and CSD analysis in particular.

for more than 60 years, current-source density (CSD) analysis has been a standard way of analyzing local field potentials (LFPs) recorded in the brain (Mitzdorf 1985; Nicholson and Freeman 1975; Pettersen et al. 2006; Pitts 1953). The CSD is easier to interpret than the LFP signals, as it is a quantification of currents entering and leaving neuronal membranes (Pettersen et al. 2012), and as effects from volume conduction ideally are absent.

The theory of CSD estimation is based on volume-conductor theory. Under the assumptions that *1*) all electrical currents in the extracellular space (ECS) are driven by electrical fields and *2*) the electrical conduction is ohmic, a Poisson-like mathematical equation can be derived for the estimation of the CSD based on simultaneous recordings of LFP in three spatial dimensions. Further, in traditional CSD analysis the variation of the CSD in the lateral directions is assumed negligible so that the estimator reduces to a double spatial derivative (Mitzdorf 1985; Nicholson and Freeman 1975; Pitts 1953). In more recently developed methods such as the iCSD (Pettersen et al. 2006) and kCSD methods (Potworowski et al. 2012), this restriction of no lateral-activity variation is lifted, but the estimators are still based on the same Poisson-like mathematical equation assuming only electrically driven currents.

However, also non-ohmic currents could in principle be present in the ECS. For example, it is well known that longer periods of intense activity may generate pronounced concentration gradients in the ECS (Kofuji and Newman 2004). Previous studies have suggested that diffusion of charged ions along extracellular gradients may influence the LFP either directly by evoking diffusion potentials (Dietzel et al. 1989; Halnes et al. 2016) or indirectly by introducing (Warburg-type) filtering effects in the impedance of the extracellular medium (Bédard and Destexhe 2009; Bédard et al. 2010). Diffusive influences on the LFP imply a violation of a key assumption behind most frameworks used in CSD analysis to date. Whereas the implications of putative filtering effects for CSD analysis have been investigated theoretically (Bédard and Destexhe 2011), no previous study has explored the effect of diffusion potentials on CSD analysis.

In the present paper we explore by means of biophysical modeling *1*) the error introduced in CSD estimates by neglecting effects from diffusion potentials and *2*) how the present CSD-analysis methods can be extended to eliminate or reduce this error. We use a previously developed computational model to simulate the ECS dynamics of the ion concentrations (*c_k_*) and potential (*V*) surrounding a small population of pyramidal neurons (Halnes et al. 2016). The model is based on the Kirchhoff-Nernst-Planck (KNP) formalism (Halnes et al. 2016) which accounts for effects of both diffusion and electrical migration on the ECS dynamics. In this in silico scenario, the true CSD (i.e., the spatiotemporal distribution of neuronal transmembrane currents) is known, and can be compared with the conventional CSD estimate based on *V*, and an alternative CSD estimate based on *V* and *c_k_* (see methods). The standard CSD estimate is found to deviate dramatically from the true CSD when extracellular concentration gradients become large, whereas the alternative CSD estimate is shown to accurately predict the true CSD (see results).

## METHODS

Below, we first present the CSD theory for the cases without and with extracellular diffusion being accounted for. Next, we present the modeling framework used to simulate the output of a neuronal population (true CSD), and the extracellular variables *V* and *c_k_* (used in CSD estimates).

### 

#### CSD estimates.

A general starting point when deriving the CSD theory is that of current conservation in the ECS (Pettersen et al. 2012):

(1) $$\nabla \cdot \overset{\to }{J}=CSD.$$

Here $\overset{\to }{J}$ is the ECS current density, and *CSD* is the volume density of cellular current sources. If the ion dynamics in the ECS is described by the Nernst-Planck equation for electrodiffusion, the net electrical current is (Halnes et al. 2013):

(2) $$\overset{\to }{J}=-\sigma \nabla V-F\underset{k}{\sum }\left(z_{k}D_{k}\nabla c_{k}\right).$$

Here, the first term on the right is the ohmic current, with σ being the extracellular conductivity. The second term is the diffusive current. The sum is taken over all ion species *k*, characterized by their concentrations (*c_k_*), valences (*z_k_*) and diffusion constants (*D_k_*). *F* is Faraday’s constant. With this expression for $\overset{\to }{J}$, *Eq. 1* becomes:

(3) $$CSD_{\text{V,c}}=-\nabla \cdot (\sigma \nabla V)-F\underset{k}{\sum }\left(z_{k}D_{k}\nabla^{2}c_{k}\right).$$

*CSD*_V,c_ represents the most complete CSD estimate considered here. In the following, we also consider two alternative CSD estimates. If we only include the first term on the right hand side of *Eq. 3*, we get

(4) $$CSD_{\text{V}}=-\nabla \cdot (\sigma \nabla V)$$

which is the classical CSD estimate from standard theory (Mitzdorf 1985; Nicholson and Freeman 1975; Pettersen et al. 2006). To neglect diffusion in CSD analysis is thus equivalent to assume that the second term on the right-hand side of *Eq. 3* is much smaller than the first term. If concentration gradients are sufficiently large, this assumption can be violated, and *CSD*_V_ will become an inaccurate CSD estimate.

We note that in the KNP simulations (used here), the ECS conductivity σ was a local function of ion concentrations, whereas σ is typically assumed to be constant in in classical CSD theory. However, concentration-dependent variations of σ were relatively small in our simulations, and in additional test simulations we verified that the choice between a constant or nonconstant σ was not important for our main results (see also Halnes et al. 2016).

For completeness, we also included a CSD estimate based solely on the last term in *Eq. 3*:

(5) $$CSD_{\text{c}}=-F\underset{k}{\sum }(z_{k}D_{k}\nabla^{2}c_{k}).$$

*CSD*_c_ is based solely on ionic diffusion and is not intended as a serious candidate for a CSD estimator. It was included because it shows in isolation the role of ionic diffusion in CSD analysis.

The CSD estimates and the true CSD (see below) were low-pass filtered with a threshold value of 500 Hz, a typical cut-off frequency for LFPs (Einevoll et al. 2013).

#### Computational model.

The ECS dynamics of *V* and *c_k_* used in the CSD analysis were computed using a previously published model (Halnes et al. 2016). Since we here used the same simulation setup as in the orginal study, we only briefly present the model below, and refer to the original work for further model details.

The model simulated the ECS dynamics surrounding a small population of 10 pyramidal neurons, based on a well-established neuron model (Hay et al. 2011) implemented in the NEURON/Python simulation environment (Hines et al. 2009). The population was driven by Poissonian synaptic input through 10,000 synapses at each neuron, uniformly distributed across the membranes. The neurons received the same input statistic during the full simulation. It took ~1 s from when the input was turned on until each individual neuron had settled into a steady-state firing activity with an average firing rate of ~5 action potentials per second, a typical firing rate for excitatory cortical neurons (de Kock and Sakmann, 2009). Focusing only on the steady-state scenario, we defined *t* = 0 to be 1.6 s after the stimulus was turned on. We simulated the extracellular dynamics (of ion concentrations and *V*) and performed CSD analysis only for *t* > 0. The simulations produced time series of fluxes of different ion species entering/leaving the ECS at different spatial locations through synaptic currents and nine different voltage- and Ca^2+^-activated ion channels. The included ion species were Na^+^, K^+^, Ca^2+^, and X^−^, where the latter represents a nonspecified anion. Since diffusion typically takes place on a long time scale, the simulated time was quite long, i.e., 84 s. Active transporters were not included in the model (Hay et al. 2011).

**Fig. 1.**
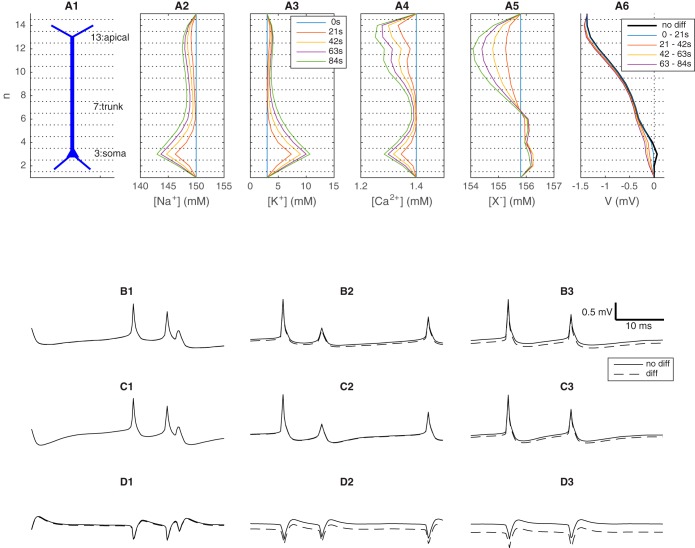
Model system. *A*: a one-dimensional piece of tissue subdivided into 15 subvolumes (depth intervals). A population of 10 neurons (only one shown) occupied the interior 13 subvolumes (*A1*). Ionic output fluxes into all compartments were recorded in an 84 s simulation. *A2*–*A5*: extracellular concentration gradients at selected time points in the simulation. *A6*: extracellular potential (*V*), low-pass filtered by taking the temporal average over the time intervals indicated in the legend. *B*–*D*: fast temporal dynamics of *V* in the ECS subvolumes containing the apical dendrites (*B*), the dendritic trunks (*C*), and the somata (*D*). Different columns show snapshots of *V* taken at different times in the simulation. Solid lines represent simulations with the full electrodiffusive formalism, whereas dashed lines represent simulations where diffusion was not included in the ECS dynamics. The legend in *A3* applies to all panels *A2*–*A5*. The scale bar in *B3* and legend in *D3* apply to all panels in *B*–*D*.

Motivated by the layered structures of brain regions such as cortex and hippocampus, we assumed lateral homogeneity (Halnes et al. 2016). The model represents a one-dimensional piece of brain tissue, meaning that variation of *V* and *c_k_* occurred only in one spatial direction, i.e., the depth direction *z*. The piece of tissue was subdivided into 15 subvolumes (hereby called voxels) along the *z*-axis, each 100 μm in height (Fig. 1*A1*). The voxel volume was *V*_vox_ = 60,000 μm^3^, and gave an ECS volume per neuron that is typical for cortical tissue (Halnes et al. 2016). The neurons were oriented so that they occupied the interior 13 voxels, while the top (*n* = 15) and bottom (*n* = 1) voxels were auxiliary compartments where *c_k_* was fixed at baseline levels. The extracellular dynamics of *c_k_* and *V* were computed with the KNP-formalism (Halnes et al. 2013; Halnes et al. 2015), and accounted for the neuronal output as well as electrical migration and diffusion of ions in the ECS (Halnes et al. 2016).

The NEURON simulation gave us the true current-source density *CSD*_true,_ defined as

(6) $$CSD_{\text{true}}=I_{\text{m}}/V_{\text{vox}},$$

where *I*_m_ denotes the total transmembrane current *I*_m_ (sum of capacitive current and all ionic currents) into the ECS of a given voxel. *CSD*_true_ could be compared with the three CSD estimates based on extracellular variables (*Eqs. 3**–**5*).

## RESULTS

Figure 1 shows the dynamics in the ECS resulting from the population activity described above. The neuronal exchange of ions with their surroundings caused ECS concentration gradients to gradually develop over time (Fig. 1, *A2–A5*). The most pronounced concentration shifts were the shifts in Na^+^ (Fig. 1*A2*) and K^+^ (Fig. 1*A3*) concentrations, which during the 84-s simulation were shifted by up to ~7 mM relative to the basal concentrations. The concentration shifts were largest surrounding the somatas (voxel *n* = 3). This was due to the action potential-generating currents (Na^+^ uptake and K^+^ release) being largest there.

### 

#### Diffusion induces shifts in extracellular potentials.

The neurodynamics evoked voltage changes in the ECS. On a slow time scale, there was an almost sustained voltage gradient across the tissue depth, as we see in Fig. 1*A6*, where *V* has been temporally averaged over the 21-s time intervals indicated in the legend. The black, thick line represents a simulation where diffusive effects on *V* were not accounted for. In this case, the average gradients in the four (21 s) time intervals were approximately identical and were indistinguishable by eye. This was because the neurons received the inputs with the same statistics throughout the simulation, so that the transmembrane current sources were effectively constant at this long time scale. Similar sustained voltage profiles that vary by a few microvolts across the depth of the cortex have been seen in experiments (see, e.g., Cordingley and Somjen 1978; Dietzel et al. 1989).

The remaining curves in Fig. 1*A6* represent a simulation where diffusive effects on *V* were accounted for. In this case, *V* was affected by the concentration gradients that developed in the system, and the average gradients in the four 21-s intervals were not identical. Outside the somatas (voxel *n* = 3), *V* was shifted by ~0.2 mV during the simulation. Such diffusion-evoked potential shifts are well known, and their genesis was thoroughly explored in Halnes et al. (2016).

Diffusion had no visible effects on the short-term fluctuations in *V*, as the raw (i.e., not temporally averaged) time series in *V* shows. Figure 1, *B*–*D* depicts snap shots of the time development of *V* at three selected locations, i.e., in a voxel containing branching apical dendrites (Fig. 1*B*), in a voxel on the trunk of the apical dendrite (Fig. 1*C*), and in the soma voxel (Fig. 1*D*). Again, we have compared *V* obtained in simulations where diffusion was included (solid lines) and not included (dashed lines). The solid and dashed lines were essentially parallel in the short time windows displayed in Fig. 1, *B1–D3*, showing that brief signals (such as AP signatures) were unaffected by the presence of diffusion. However, we again see that diffusion shifted *V* slowly from early in the simulation where the solid and dashed lines coincided (Fig. 1, *B1*, *C1*, and *D1*) toward the end of the simulation where *V* had a more negative value in simulations where diffusion was included (Fig. 1, *B3, C3*, and *D3*). The diffusion-evoked shifts in *V* were most pronounced in the soma voxel (Fig. 1*D3*), since the concentration gradients were steepest there, but could also be seen at the other locations (Fig. 1, *B3* and *C3*).

The diffusion-induced shifts of *V* occurred on the same slow time scale as the shifts of *c_k_* (Fig. 1, *A2–A5*). The gradually increasing diffusive currents were thoroughly investigated previously (Halnes et al. 2016) and will not be further explored here. In the following we rather use the simulation results summarized in Fig. 1 to study the implication the diffusion-evoked effects on *V* has for CSD analysis.

#### CSD analysis.

The standard equation in CSD analysis is *Eq. 4*, which implicitly assumes that electrical currents due to extracellular diffusion are negligible, and that *V* exclusively reflect ohmic currents. Here, we explore the errors induced by making this assumption.

**Fig. 2.**
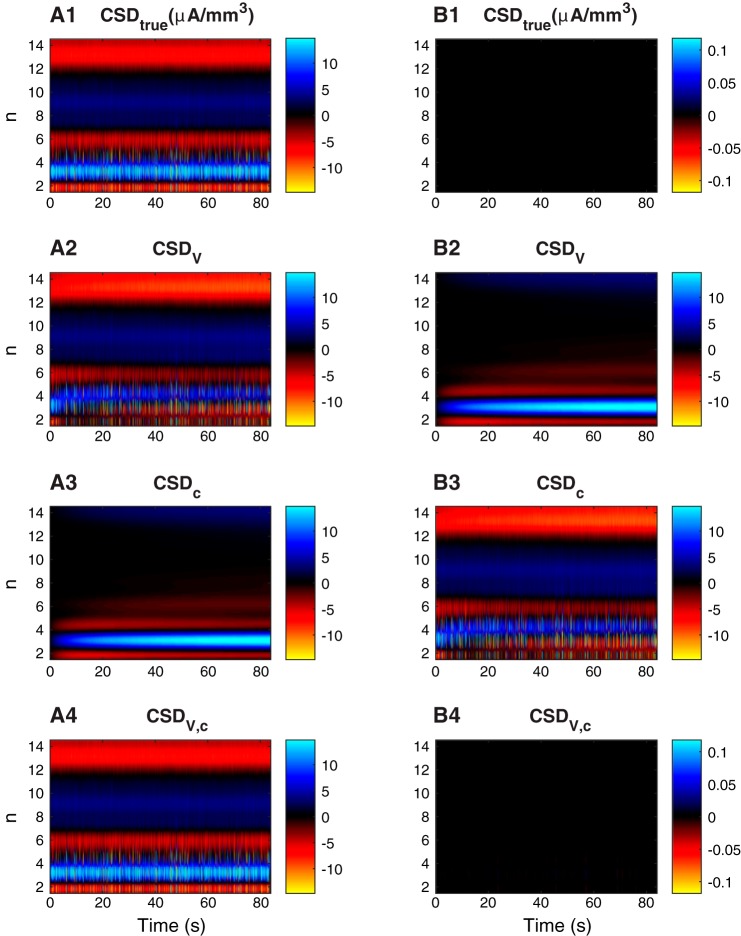
Current-source density. *A*: CSD at different depth levels (*y*-axis) as a function of time (*x*-axis). *A1*: true CSD. *A2*: standard CSD estimate based only on the extracellular potential. *A3*: CSD estimate based only on extracellular ion concentrations. *A4*: complete CSD estimate based on extracellular potential and ion concentrations. *B2*–*B4*: error relative to true CSD (ΔCSD = CSD_true_ − CSD_estimate_). *B1*: the error in the true CSD is by definition zero. In all panels, the CSD was low-pass filtered with a threshold value of 500 Hz, a typical cutoff frequency for local field potentials (Einevoll et al. 2013). Units in all panels are μA/mm^3^.

Figure 2 shows the CSD for the previously shown simulation (Fig. 1). Figure 2*A1* shows the true CSD, i.e., the true distribution of neuronal current sources as given in *Eq. 6*. The neurons received input with the same statistics throughout the simulation, meaning that the steady-state firing patterns and the CSD remained roughly constant at the long time scale depicted here. Figure 2, *A2–A4*, shows the CSD estimates based on *Eqs. 4*, *5*, and *3*, respectively whereas Fig. 2, *B2–B4*, shows the error in the different CSD estimates relative to the true CSD (Fig. 2*B1* illustrates the error in the true CSD which by definition is zero).

Although the CSD was low-pass filtered, it still contained signatures of neuronal action potentials, as seen by the brief (bright) pulses in Fig. 2, *A1, A2*, and *A4*. However, as diffusion is a slow process, we are here more interested in the lower frequencies of the CSD, i.e., the enduring, smoother signals that appear in the background. By comparing Fig. 2, *A1* and *A2*, we see that the true CSD (*CSD*_true_) and the standard CSD estimate (*CSD*_V_) were not identical. In accordance with the observed ECS concentration profiles (Fig. 1*A*), the error was largest outside the somata, and built up at the time course of tens of seconds (*n* = 3 in Fig. 2*B2*).

As expected, the CSD estimate based solely on ECS ion concentrations (*CSD*_c_) gave poor predictions of the CSD at most depths (Fig. 2*B3*). However, as noted above, *CSD*_c_ was not intended as a serious candidate for a CSD estimator. It was included because it was identical to the error in the standard CSD estimate Δ*CSD*_V_ (compare Fig. 2, *B2* and *A3*). This illustrates that the source of the error Δ*CSD*_V_ was the missing contribution from ionic diffusion. An improved CSD estimate was thus obtained simply by adding the diffusive contribution to the standard CSD estimate: *CSD*_V,c_ = *CSD*_V_ + *CSD*_c_. As we see in Fig. 2*A4*, the improved estimate *CSD*_V,c_ accurately estimated the true CSD.

The interplay between diffusive currents and *V* can be understood from the principle of charge conservation. According to the true CSD (Fig. 2*A1*), the somata represented the main current source (blue shading at *n* = 3), while there were current sinks along the trunk and in the apical dendrites (red shading in *n* = 6, 13, and 14). This source/sink configuration was essentially preserved throughout the simulation, and uniquely determined the ECS current running from sources to sinks to complete the current loop. The ECS current contained an ohmic and a diffusive term, so that *I* = *I*^ohmic^ + *I*^diff^. Early in the simulation the concentration gradients were small, so that *I* ~ *I*^ohmic^, and the standard estimate (*CSD*_v_) predicted the true CSD accurately for small times *t* (Fig. 2*B2*). However, as concentration gradients built up, *I*^diff^ increased, and *I*^ohmic^ had to decrease correspondingly for the net current to remain constant. Since *I*^ohmic^ = −σ*dV*/*dz*, the effect of this was a reduction of the ECS potential gradients, as we saw in Fig. 1*F*. Hence, diffusion can cause *V* to change even in scenarios where the true CSD is constant.

#### Spurious current sources in the presence of diffusion.

**Fig. 3.**
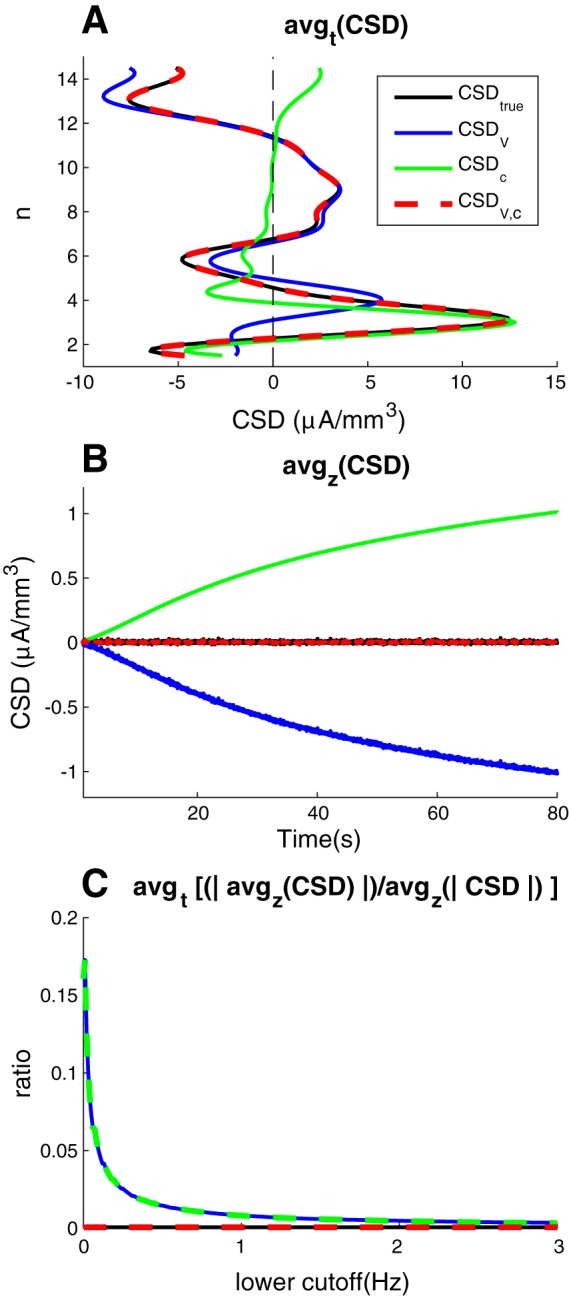
Average current-source density. *A*: the spatial distribution of the temporally averaged CSD estimates, *avg_t_*(*CSD*). *B*: the temporal development of the spatially averaged CSD estimates, *avg_z_*(CSD). *C*: temporally averaged net (spurious) current monopole erroneously estimated in system as function of lower cutoff frequency, plotted relative to the absolute values of the CSD, *avg*_t_ [|*avg*_z_(CSD)|/*avg*_z_(|CSD|)]. Upper cutoff frequency was 500 Hz in all panels. Lower cutoff frequency was 0 in *A* and *B* and varied from 0 to 3 Hz in *C*.

An investigation of the spatial and temporal averages of the CSD signal may be useful for extracting some key points from the above analysis. Confirming what we saw above, the temporal averages (Fig. 3*A*) show that the improved CSD estimate *CSD*_V,c_ (red line) was essentially identical to the estimated true CSD (black line), whereas the standard estimate *CSD*_V_ (blue line) gave a large error outside the somata (*n* = 3). In addition, the temporal averages illustrate clearly that *CSD*_V_ wrongly estimated the depth position of the main current source. While the true CSD peaked at the somata (*n* = 3), *CSD*_V_ peaked in voxel *n* = 4, which was 100 μm higher up. Again, the explanation lies in the steep concentration gradients surrounding the somata, which caused a large proportion of the ECS charge transport away from the soma to be diffusive, and thus not registered by *CSD*_V._ For the same reason, the location of the main current source was better predicted by the concentration-based estimate *CSD*_c_ (green line). Of course, these findings were specific to the particular model used here, but illustrate how standard CSD analysis can become misleading in the presence of steep concentration gradients.

The spatial average of the CSD (Fig. 3*B*) illustrates another important point. Since neurons form closed membranes, charge conservation demands that the sum of all currents entering/leaving should be zero at all times. The spatial mean of the true CSD was therefore always zero (black line). However, this zero-sum was not predicted by the standard estimate *CSD*_V_ (blue line). According to *CSD*_V,_ the neuronal population appeared as a spurious monopolar current sink which increased in magnitude throughout the simulation. Oppositely, according to the diffusion-based measure (*CSD*_c_), the neuronal population appeared as a spurious monopolar current source. As shown by the improved CSD estimate *CSD*_V,c_ (red line), these spurious sources/sinks were perfectly balanced, so that the spatial mean of the CSD was always zero. We conclude that the presence of ECS diffusion, if not properly accounted for in CSD analysis, may lead to the prediction of spurious current monopoles.

In the results shown so far, we have kept the full frequency range (from 0 to 500 Hz) of the CSD. However, being a slow process, diffusion predominantly influenced the low-frequency components of the CSD. Diffusive effects were therefore dramatically reduced when the lowest frequency components were filtered out from the CSD signal. Figure 3*C* shows how the diffusion-induced spurious current sources (seen in Fig. 3*B*) decreased steeply as a function of the lower cutoff frequency of the CSD. Thus, for the present example, effects of diffusion on the CSD estimate appears small for frequency components above a few hertz. A limitation of concentration-dependent effects to frequency components up to maximally a few hertz has also been estimated previously, but then from experimentally observed time courses of ion dynamics, and without modeling diffusion explicitly (Gratiy et al. 2017).

## DISCUSSION

In standard CSD analysis, the CSD is assumed to be given by a Laplace-like operator acting on the ECS potential (cf. *Eq. 4*), reducing to a double-spatial derivative along the depth direction if a constant electrical conductivity and no lateral variation in the LFP is assumed (Mitzdorf 1985; Nicholson and Freeman 1975; Pettersen et al. 2006). As shown here, the standard CSD estimate becomes inaccurate when extracellular concentration gradients become large. The explanation lies in diffusion-induced changes in the extracellular potential *V*, which are not accounted for in the standard CSD analysis. We therefore proposed an improved CSD estimator which accounts for diffusive effects (*Eq. 3*) and showed that it gave correct CSD estimates in simulations where the true CSD was known. Diffusive effects were found to be most relevant for the low-frequency components of the CSD.

By design, this study was performed in two steps. First, we simulated the output from a small population of pyramidal neurons and defined this as the true CSD. Second, we simulated the extracellular dynamics resulting from this output, and compared the performance of CSD estimators that did and did not account for extracellular diffusion in terms of their success in estimating the true CSD. As for the first step, we used a previously developed model of a small population of pyramidal neurons, designed so that ion concentrations changed locally by up to 7–8 mM during the simulation (Halnes et al. 2016). Concentration changes in this range have been observed experimentally under nonpathological conditions (Kofuji and Newman 2004; Singer and Lux 1975) but may be more generally representative for pathological conditions involving seizures (Fröhlich et al. 2008; Raimondo et al. 2015). In the simulations presented here, they occurred under steady-state population dynamics. Consisting of only a single neuron species and lacking neuronal and glial uptake mechanisms, the model was admittedly too simple to represent any specific biological system in detail. However, the main focus in this study was on the second step, and as diffusive effects on CSD estimates depended mainly on extracellular concentration gradients (and not on their origin), the main conclusions are unlikely to depend on these model choices. For further discussion on how model-specific choices could influence the simulated extracellular dynamics, we refer to the original publication (Halnes et al. 2016).

The diffusion potentials addressed here are those arising along large-scale concentration gradients. Diffusion potentials of this kind are well known in electrolyte theory, and are often referred to as liquid junction potentials, since they are most pronounced at the boundary between two solutions of different ion composition (Aguilella et al. 1987; Perram and Stiles 2006). These diffusion potentials should not be confused with the Warburg-type filtering effects hypothesized by Bedard and Destexhe (with coworkers) to arise due to diffusive effects at neuronal membranes when charge is transferred from the intracellular to the extracellular space (Bédard et al. 2010; Bédard and Destexhe 2011; Gomes et al. 2016]. The Warburg-type filtering effects describe a phenomenon that is different from and complementary to the diffusion potentials explored in the current study.

The findings made here could be of relevance for interpreting the experimental recordings by Riera et al. (2012). Using the standard CSD estimate (*CSD*_V_), they found that the CSD did not sum to zero over the volume of a cortical barrel column. Seemingly, this indicated the presence of a non-zero current-source monopole on a mesoscopic (cell population) scale. The possible origin of these apparent current monopoles was later debated (Bédard and Destexhe 2013; Destexhe and Bedard 2012; Gratiy et al. 2013; Riera and Cabo 2013). One (of several) possible explanations is that they could be spurious monopoles reflecting the presence of diffusive effects that were not accounted for in the analysis (as in Fig. 3*B*). It should be noted, however, that the monopolar contributions seen in the experiments were claimed to sum to zero over time. A temporal balancing of sources and sinks is not an impossibility, but neither a requirement for the diffusion-evoked, spurious monopoles that we explored in Fig. 3*B*. This may also indicate that effects other than those studied here could be in play in the experiments by Riera et al. (2012).

In conclusion, *Eq. 3* accounts for diffusive effects on *V* and represents an improvement of the CSD theory. By prediction, diffusion effects occur at low frequencies and would predominantly be relevant for interpreting signals for which the main frequency component is not much higher than 1 Hz, such as slow neocortical rythms or delta waves. Unfortunately, application of the improved theory in principle requires explicit knowledge not only of *V*, but also of the extracellular concentrations *c_k_* of all involved ion species. To record all these data simultaneously may be experimentally challenging. However, our approach does offer a means to predict whether diffusive effects are likely to be present in a given experimental condition. First, we have previously predicted that when diffusion effects are dominant, the LFP power spectrum should express a 1/*f*^2^ decay for lower frequencies (between 0 and 1 Hz) (Halnes et al. 2016), which could be readily verified by making measurements over long time periods. Second, the results in Fig. 3*C* lead to another testable prediction, i.e., that if diffusion effects are present, standard CSD analysis should estimate a (spurious) monopolar population current source, which should vanish if the lower cutoff frequency in the LFP recordings are increased to frequencies much higher than 1 Hz.

## GRANTS

This project was funded by the European Union Seventh Framework Programme (FP7/2007–2013) under grant agreement 604102 (Human Brain Project, HBP), and the Research Council of Norway (ISP, BIOTEK2021/Digital Life).

## DISCLOSURES

No conflicts of interest, financial or otherwise, are declared by the authors.

## AUTHOR CONTRIBUTIONS

G.H. and G.T.E. conceived and designed research; G.H. and T.M.-M. performed experiments; G.H., K.H.P., and G.T.E. analyzed data; G.H., K.H.P., and G.T.E. interpreted results of experiments; G.H. prepared figures; G.H. drafted manuscript; G.H., T.M.-M., K.H.P., O.A.A., and G.T.E. edited and revised manuscript; G.H., T.M.-M., K.H.P., O.A.A., and G.T.E. approved final version of manuscript.
